# Counteracting gemcitabine+nab-paclitaxel induced dysbiosis in KRAS wild type and KRAS^G12D^ mutated pancreatic cancer in vivo model

**DOI:** 10.1038/s41420-023-01397-y

**Published:** 2023-04-05

**Authors:** Concetta Panebianco, Federica Pisati, Annacandida Villani, Annapaola Andolfo, Marynka Ulaszewska, Edoardo Bellini, Carmelapia Ferro, Renato Lombardi, Fabrizio Orsenigo, Tiziana Pia Latiano, Beatrice Belmonte, Claudio Tripodo, Francesco Perri, Valerio Pazienza

**Affiliations:** 1 Division of Gastroenterology, Fondazione IRCCS Casa Sollievo della Sofferenza, Viale dei Cappuccini, 1, 71013 San Giovanni Rotondo, FG Italy; 2 Histopathology Unit, Cogentech S.C.a.R.L, FIRC Institute of Molecular Oncology (IFOM), Via Adamello, 16, 20139 Milan, MI Italy; 3 Proteomics and Metabolomics Facility (ProMeFa), IRCCS San Raffaele Scientific Institute, Via Olgettina, 60, 20132 Milan, Italy; 4 Unit of Pharmacy, Department of Pharmaceuticals, IRCCS Casa Sollievo della Sofferenza, San Giovanni Rotondo, Italy; 5 IFOM, FIRC Institute of Molecular Oncology, Milan, Italy; 6 Oncology Unit Fondazione IRCCS Casa Sollievo della Sofferenza, Viale dei Cappuccini, 1, 71013 San Giovanni Rotondo, FG Italy; 7 Tumor Immunology Unit, Department of Health Promotion, Mother and Child Care, Internal Medicine and Medical Specialties “G. D’Alessandro”, University of Palermo, Via del Vespro 129, 90127 Palermo, Italy

**Keywords:** Cancer models, Chemotherapy

## Abstract

Pancreatic cancer (PC) has a very low survival rate mainly due to late diagnosis and refractoriness to therapies. The latter also cause adverse effects negatively affecting the patients’ quality of life, often requiring dose reduction or discontinuation of scheduled treatments, compromising the chances of cure. We explored the effects of a specific probiotic blend on PC mice xenografted with KRAS wild-type or KRASG12D mutated cell lines alone or together with gemcitabine+nab-paclitaxel treatment to then assess tumor volume and clinical pathological variables. Beside a semi-quantitative histopathological evaluation of murine tumor and large intestine samples, histochemical and immunohistochemical analyses were carried out to evaluate collagen deposition, proliferation index Ki67, immunological microenvironment tumor-associated, DNA damage markers and also mucin production. Blood cellular and biochemical parameters and serum metabolomics were further analyzed. 16S sequencing was performed to analyze the composition of fecal microbiota. Gemcitabine+nab-paclitaxel treatment impaired gut microbial profile in KRAS wild-type and KRASG12D mice. Counteracting gemcitabine+nab-paclitaxel- induced dysbiosis through the administration of probiotics ameliorated chemotherapy side effects and decreased cancer-associated stromatogenesis. Milder intestinal damage and improved blood count were also observed upon probiotics treatment as well as a positive effect on fecal microbiota, yielding an increase in species richness and in short chain fatty acids producing- bacteria. Mice’ serum metabolomic profiles revealed significant drops in many amino acids upon probiotics administration in KRAS wild-type mice while in animals transplanted with PANC-1 KRASG12D mutated all treated groups showed a sharp decline in serum levels of bile acids with respect to control mice. These results suggest that counteracting gemcitabine+nab-paclitaxel-induced dysbiosis ameliorates chemotherapy side effects by restoring a favorable microbiota composition. Relieving adverse effects of the chemotherapy through microbiota manipulation could be a desirable strategy in order to improve pancreatic cancer patients’ quality of life and to increase the chance of cure.

## Introduction

With a 5-year survival rate of only 10%, pancreatic cancer is among the malignancies with the lowest survival [1]. Particularly, pancreatic ductal adenocarcinoma (PDAC), which accounts for more than 90% of pancreatic cancers [2, 3], owes its poor prognosis to late diagnosis and refractoriness to available therapies [4, 5]. Since 1997 gemcitabine monotherapy has represented the standard of care for unresectable PDAC until 2013, when its combination with nanoparticle albumin- bound paclitaxel (nab-paclitaxel) was unveiled more effective in increasing overall survival, progression-free survival, and response rate, though presenting greater toxicity in terms of myelosuppression and peripheral neuropathy [6]. Besides negatively affecting patients’ quality of life, chemotherapy-related adverse effects may require dose reduction or discontinuation of planned treatment, which further hinder the chances of cure. In the light of this view, finding out supportive approaches to conventional treatments with the ability to improve efficacy and limit toxicity, would be highly desirable. Accumulating evidence has shown that gut microbiota plays a pivotal role in modulating both efficacy and toxicity of anticancer therapies, mainly due to the ability of bacteria to metabolize drugs and produce biologically active compounds with immunomodulatory properties [7]. Diet, consumption of prebiotics and probiotics are just some feasible strategies to manipulate the gut microbiota composition, which could likely turn out in a better response to treatments [7, 8].

A recent meta-analysis demonstrated that probiotics intake in patients with non-small cell lung cancer, improves the efficacy of immune-checkpoint inhibitors treatment [9], supporting the rationale that intervening on gut microbiota could result in maximizing the clinical benefits to patients. We previously demonstrated that administering a specific probiotics blend in a BxPC-3 xenografted mouse model of PDAC, treated or not with gemcitabine monotherapy, resulted in reduced stroma deposition and increased cell apoptosis within the tumor, preservation of the intestinal villi integrity, increased mucin production and proliferative activity of crypt epithelial cells, reduction of chemotherapy-related hematologic toxicity. All these beneficial effects were accompanied by modifications in gut microbiota with an enrichment in anti-inflammatory bacteria and a higher species richness [10].

In the present study, we aimed to investigate the impact of the aforementioned probiotics mixture on two mouse models subcutaneously injected with the human PDAC BxPC-3 and PANC-1 cell lines. BxPC-3 and PANC-1, which differ from each other for many genotypic and phenotypic aspects [11], were chosen to represent, respectively, PDAC with wild-type and mutated (G12D) KRAS, which is the gene most frequently altered (>95% of cases) in such disease [12]. The effects of probiotics supplementation to the gemcitabine+nab-paclitaxel chemotherapy regimen were also investigated in both animal models.

## Results

### Effect of chemotherapy and/or probiotic administration on pancreatic tumor growth and histology in mice

**Fig. 1:**
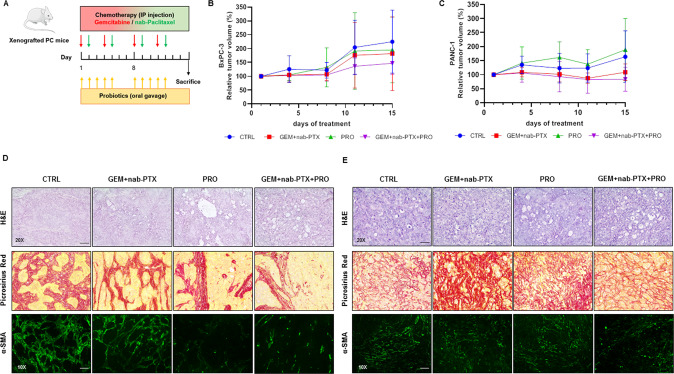
Impact of chemotherapy and/or probiotic treatment on mice pancreatic cancer growth and histology. Scheme of the treatment with Gemcitabine+nab-Paclitaxel and/or probiotics in xenografted pancreatic cancer mice (**A**). Growth curves obtained by measuring tumor volumes in BxPC-3 (**B**) and PANC-1 (**C**) bearing mice belonging to the different experimental groups. Data of relative tumor volumes are shown as mean ± SD. Representative histological stain of H&E (20X magnification), Picrosirius Red (×20 magnification) and α-SMA (×10 magnification) in BxPC-3 (**D**) and PANC-1 (**E**) tumor sections. Scale bar 50 µm.

The tumor volume of treated mice as summarized in Fig. 1A was monitored through all the experimental protocol. In mice treated with chemotherapy alone and chemotherapy plus probiotics, tumor volumes at the end of the protocol trended down, without reaching statistical significance in either BxPC-3 (Fig. 1B) and PANC-1 (Fig. 1C) bearing mice, due to the 2-weeks short treatment period thus considering only four cycles of chemotherapy.

However, in both BxPC-3 (Fig. 1D) and PANC-1 (Fig. 1E) animals, histopathological evaluation, performed on H&E stained sections, highlighted a higher number of vacuoles in all treatment groups (especially in those receiving probiotics) as compared to the control. In BxPC-3 tumor sections, Picrosirius Red staining revealed a dense collagen deposition surrounding the tumor cell foci in CTRL, which was loosened upon the three treatments. Moreover, immunohistochemistry for α-smooth muscle actin (α-SMA), used as a marker of myofibroblasts and vascular mural cells, showed a decreased contribution of these elements in the packagement for stromal bundles in all three treatments. A quantitative evaluation of Picrosirius Red (Fig. 1A) and alpha-SMA (Fig. S1B) staining showed statistically significant decrease in all three treatments when compared to control, with a more pronounced effect observed in the two groups supplemented with probiotics. Both stainings also significantly decreased in combined treatment with respect to chemotherapy alone. Similar results were observed in PANC-1 xenografted mice, in which the highest deposition of collagen fibers was detected upon chemotherapy alone; collagen stained with Picrosirius (Fig. S1D) and alpha-SMA positivity (Fig. S1E) were significantly decreased in PRO as compared to CTRL and in GEM + nab-PTX + PRO as compare to GEM + nab-PTX.

**Fig. 2:**
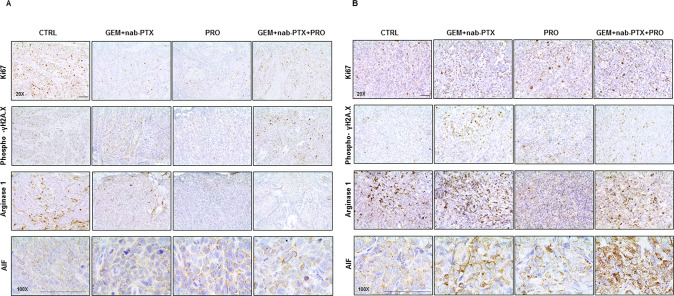
Immunophenotypical characteristics of mice pancreatic cancer under chemotherapy and/or probiotic treatment. Representative immunohistochemical analyses of Ki67 (×20 magnification), Phospho- γH2A.X (×20 magnification), Arginase 1 (×20 magnification), and AIF (×10 magnification) in BxPC-3 (**A**) and PANC-1 (**B**) tumor sections. Scale bar 50 µm.

As represented in Fig. 2A, in BxPC-3 tumor samples, a decreased expression of the proliferation index Ki67 was observed in treated groups as compared to CTRL. A higher expression of both Phospho-H2A.X (Figure S1C) and AIF (used as a marker for DNA damage and apoptosis, respectively) was detected in combined treatment (GEM + nab-PTX + PRO), with respect to the other groups. Furthermore, the same groups highlighted the lowest expression of the Arginase-1 positive pro-tumoral M2-polarized macrophages. Regarding PANC-1 tumor specimens (Fig. 2B), contrariwise, no significantly difference of Ki67 expression was observed among the groups; both Phospho-H2A.X (Fig. S1F) and AIF showed a higher expression in GEM + nab-PTX + PRO and in GEM + nab-PTX groups, with respect to the other groups. PRO and GEM + nab-PTX + PRO also revealed a lower expression of Arginase-1, as compared to their respective controls.

### Effect of chemotherapy and/or probiotic administration on animal hematological and biochemical parameters

We next evaluated whether probiotics administration could have a beneficial effect against chemotherapy-related toxicity. To this purpose, hematological and biochemical parameters were assessed in both BxPC-3 and PANC-1 xenografted mice at the end of the treatment (Table 1). In BxPC-3 bearing mice treated with only GEM + nab-PTX showed a significantly lower number of white blood cells as compared to CTRL, whereas no difference was observed when probiotics were supplemented (GEM + nab-PTX + PRO). On the contrary, both chemotherapy-receiving groups had a lower number of neutrophils with respect to CTRL. Moreover, GEM + nab-PTX treatment induced a significant decrease in platelet number, which was restored by probiotics supplementation to chemotherapy. Similarly, also the plateletcrit was significantly increased in combined treatment as compared to chemotherapy alone. In addition, concerning biochemical serum markers, a significant reduction in urea levels was observed in all three treatment groups in comparison with the untreated control. As for PANC-1 bearing mice, among the experimental groups the hepatic marker alkaline phosphatase (ALP) was significantly increased upon chemotherapy (GEM + nab-PTX) but not in combined treatment (GEM + nab-PTX + PRO) when compared to CTRL.

**Table 1:** Blood cell count and serum biochemistry in BxPC-3 and PANC-1 -bearing mice.

BxPC-3- bearing mice					PANC-1- bearing mice			
CTRL	GEM + nab-PTX	PRO	GEM + nab-PTX + PRO		CTRL	GEM + nab-PTX	PRO	GEM + nab-PTX + PRO
9.30 (±0.39)	8.99 (±1.50)	9.36 (±0.50)	8.56 (±0.52)**	RBC (x10^6^/µL)	8.90 (±1.10)	8.48 (±1.40)	8.56 (±1.19)	7.63 (±1.50)
14.30 (±0.47)	13.74 (±2.28)	14.24 (±0.71)	12.88 (±0.67)**	Hgb (g/dL)	13.87 (±1.44)	13.26 (±1.70)	13.09 (±1.80)	12.14 (±2.07)
43.50 (±1.91)	41.23 (±6.91)	42.81 (±2.51)	40.02 (±2.57)**	HCT (%)	41.18 (±4.00)	39.64 (±4.72)	39.14 (±5.53)	36.60 (±7.68)
46.79 (±1.62)	45.89 (±1.86)	45.78 (±1.97)	46.76 (±1.21)	MCV (fL)	46.65 (±5.49)	47.08 (±2.61)	45.75 (±2.63)	48.01 (±5.63)
15.34 (±0.43)	15.28 (±1.02)	15.19 (±0.72)	15.03 (±0.62)	MCH (pg)	15.60 (±1.51)	15.70 (±0.78)	15.28 (±0.99)	15.98 (±1.38)
14.29 (±0.16)	14.43 (±0.58)	14.46 (±0.34)	14.92 (±0.45)	RDW (%)	13.72 (±0.99)	14.52 (±0.50)	14.45 (±0.72)	14.34 (±0.91)
6.71 (±2.91)	4.03 (±1.45)*	5.83 (±1.70)	5.69 (±2.48)	WBC (x10^3^/µL)	5.33 (±2.74)	3.50 (±1.84)	4.43 (±2.33)	3.36 (±1.81)
3.60 (±1.66)	1.46 (±0.74)**	2.89 (±0.82)	1.97 (±0.88)*	Segmented neutrophils	2.40 (±1.03)	1.35 (±0.75)	2.21 (±0.77)	1.34 (±0.66)*
2.66 (±1.41)	2.23 (±0.85)	2.34 (±0.74)	3.26 (±1.63)	Lymphocytes	2.71 (±2.10)	1.94 (±1.17)	1.98 (±2.02)	1.81 (±1.19)
0.09 (±0.06)	0.08 (±0.04)	0.27 (±0.38)	0.09 (±0.10)	Monocytes	0.10 (±0.06)	0.10 (±0.09)	0.12 (±0.07)	0.10 (±0.08)
0.16 (±0.07)	0.14 (±0.12)	0.25 (±0.13)	0.24 (±0.15)	Eosinophils	0.10 (±0.06)	0.10 (±0.04)	0.09 (±0.04)	0.09 (±0.04)
0.20 (±0.19)	0.14 (±0.16)	0.09 (±0.10)	0.13 (±0.12)	Basophils	0.02 (±0.04)	0.02 (±0.04)	0.02 (±0.03)	0.00 (±0.01)
965.57 (±139.30)	675.22 (±264.18)*	962.13 (±205.62)	997.11 (±235.80)#	PLT (x10^3^/µL)	983.67 (±385.84)	853.60 (±229.06)	918.50 (±312.94)	881.67 (±370.94)
7.04 (±0.74)	7.62 (±1.29)	7.25 (±1.53)	6.99 (±0.40)	MPV (fl)	7.67 (±1.25)	7.92 (±0.94)	7.96 (±1.16)	8.12 (±0.75)
0.68 (±0.11)	0.51 (±0.19)	0.70 (±0.22)	0.70 (±0.16)#	PCT (%)	0.79 (±0.46)	0.67 (±0.18)	0.72 (±0.27)	0.72 (±0.33)
19.29 (±1.13)	17.74 (±3.65)	19.03 (±1.93)	17.61 (±2.12)	PDW (%)	14.03 (±3.90)	16.82 (±4.51)	15.49 (±3.88)	15.53 (±3.23)
148.86 (±53.54)	322.33 (±506.86)	114.00 (±44.20)	201.56 (±206.24)	AST (U/L)	689.43 (±562.67)	737.67 (±908.67)	426.38 (±460.33)	310.11 (±331.03)
74.14 (±44.61)	111.56 (±120.31)	50.13 (±31.91)	98.78 (±110.05)	ALT (U/L)	416.57 (±415.33)	343.00 (±616.65)	301.88 (±444.00)	146.11 (±276.38)
130.00 (±44.79)	149.22 (±34.49)	123.00 (±24.88)	157.11 (±43.56)	ALP (U/L)	123.71 (±41.40)	175.33 (±20.91)*	134.25 (±37.90)	148.11 (±48.26)
16.86 (±12.90)	11.00 (±6.02)	11.88 (±5.11)	18.56 (±5.79)	GGT (U/L)	14.29 (±5.77)	24.00 (±12.74)	19.25 (±10.74)	14.78 (±6.63)
71.14 (±10.30)	58.44 (±9.98)*	57.50 (±6.00)**	57.89 (±11.66)*	Urea (mg/dL)	58.86 (±13.61)	68.83 (±13.26)	56.75 (±20.93)	57.56 (±11.70)
0.85 (±0.34)	0.87 (±0.34)	0.78 (±0.35)	0.76 (±0.28)	Creatinine (mg/dL)	0.80 (±0.26)	0.92 (±0.31)	0.85 (±0.23)	0.83 (±0.24)
11.23 (±1.42)	10.97 (±1.97)	10.33 (±0.82)	10.82 (±3.27)	Phosphorus (mg/dL)	10.40 (±1.31)	10.95 (±1.84)	10.26 (±1.38)	9.26 (±1.39)

Pairwise *t*-test were performed for each parameter. Results were considered significant versus CTRL when *p < 0.05 or **p < 0.01, and significant versus GEM + nab-PTX when #*p* < 0.05.

*Significant vs. CTRL, *p* < 0.05.

**Significant vs. CTRL, *p* < 0.01.

#Significant vs. GEM + nab-PTX, *p* < 0.05.

### Effect of chemotherapy and/or probiotic administration on animal hematopoiesis

**Fig. 3:**
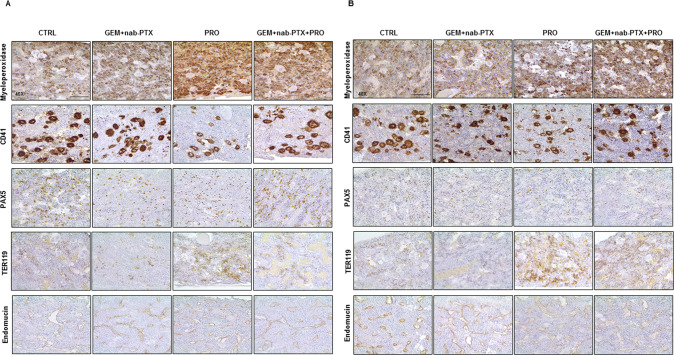
Chemotherapy and/or probiotic treatment induce changes on mice hematopoiesis. Representative microphotographs of immunohistochemistry for Myeloperoxidase, CD41, PAX5, Ter119, and Endomucin in BxPC-3 (**A**) and PANC-1 (**B**) bone marrow sections. All pictures were taken at ×40 magnification. Scale bar 50 µm.

In order to investigate the possible mechanism of the impaired blood count observed among the different groups by chemotherapy particularly in BxPC-3 bearing mice, an immunophenotypical analysis of the bone marrow hematopoietic populations was carried out. As represented in Fig. 3A, in the bone marrow of BxPC-3 animals, probiotics alone and combined with chemotherapy increased the density of granulocytes precursors expressing myeloperoxidase. Immunostaining for CD41 highlighted megakaryocyte presenting an enlargement and irregularity of nuclei with slight dysplastic morphology and with tendency to aggregate in dense clusters in groups without probiotics treatment, compared to others one. An interesting drop in B-lymphocytes, as indicated by PAX5 marker, was observed in GEM + nab-PTX group, which however was restored when chemotherapy was accompanied by probiotic supplementation. TER119 also revealed a decrease of the density of erythroid cells upon chemotherapy alone and instead an increase in probiotics intake groups. Moreover, an increased vascular density revealed by the endothelial marker endomucin was observed in probiotics-treated groups, suggesting a role in supporting the growth and proliferation. Interestingly, despite no significant effect on circulating cells, relevant differences among the groups were observed in bone marrow from PANC-1 bearing mice (Fig. 3B). In detail, similar to BxPC-3, megakaryocytes presented no significant cytological atypia upon probiotics treatment; the overall frequency of PAX5-positive B-lymphoid elements was reduced in GEM + nab-PTX condition compared to CTRL, but restored in probiotics-receiving groups; erythroid cells and vessels were also increased following probiotics administration. On the contrary, no relevant differences in myeloperoxidase-positive myeloid cells was detected.

### Effect of chemotherapy and/or probiotic administration on animal intestinal structure

**Fig. 4:**
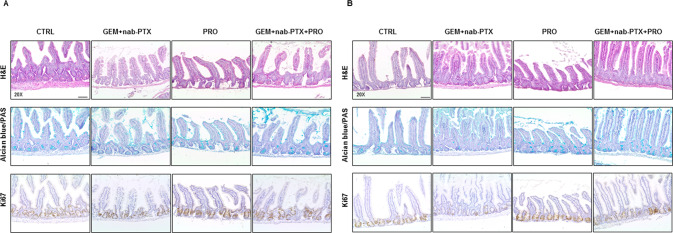
Role of chemotherapy and/or probiotic treatment on murine small intestine architecture. Representative pictures of hematoxylin/eosin, Alcian Blue/PAS and Ki67 in BxPC-3 (**A**) and PANC-1 (**B**) small intestine sections (×20 magnification). Scale bar 50 µm.

Since chemotherapy is frequently reported to damage gut mucosa and probiotics are known to have beneficial effects on intestinal health, we then performed histological analyses of mouse intestinal sections from both BxPC-3 (Fig. 4A) and PANC-1 (Fig. 4B) bearing mice. In both animal models, the integrity of villi was clearly better preserved, the mucin production as well as the number of Ki67-positive proliferating cells in the crypts were increased in probiotics-receiving mice with respect to their relative controls.

### Effect of chemotherapy and/or probiotic administration on microbiota composition in mice

**Fig. 5:**
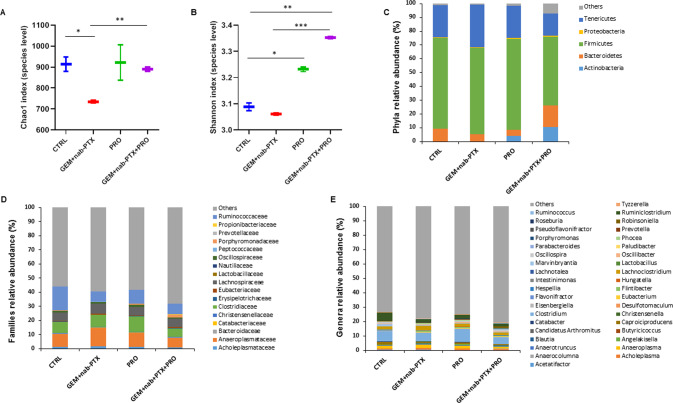
Effect of chemotherapy and/or probiotic treatment on gut microbiota alpha-diversity and taxonomic composition in BxPC-3 bearing mice. Boxplots representing species-level richness expressed by Chao1 index (**A**) and diversity expressed by Shannon Index (**B**) in the four experimental groups. Data are expressed as means of two fecal pools per group. Differences were considered significant when *p < 0.05, **p < 0.01, or ***p < 0.001. Stacked barplots showing the mean relative abundance of gut bacterial phyla (**C**), families (**D**), and genera (**E**) level. Data are expressed as means of two fecal pools per experimental group.

We next explored the impact of chemotherapy and probiotics administration on animal gut microbiota. To this purpose, fecal pools for each experimental condition underwent 16S rRNA gene sequencing. Concerning animals bearing BxPC-3 tumors, a total of 453,386 high-quality read pairs were obtained, with an average of 56,671 read pairs per sample. To analyze the within-sample diversity of the bacterial communities, alpha-diversity indices were calculated at the species level. In these mice, GEM + nab-PTX chemotherapy caused a significant drop in microbial species richness as expressed by Chao1 index, which was restored in GEM + nab-PTX + PRO group (Fig. 5A). Also diversity, represented by Shannon index, was significantly increased in both groups receiving probiotics when compared to CTRL and GEM + nab-PTX groups (Fig. 5B). When considering the microbiota composition at the phylum level (Fig. 5C), *Firmicutes* significantly decreased in both chemotherapy-receiving groups (62.3% in GEM + nab-PTX and 49.7% in GEM + nab-PTX + PRO) compared to CTRL (65.9%). *Bacteroidetes*, instead, were significantly reduced upon chemotherapy alone (5.5% in GEM + nab-PTX vs. 9.1% in CTRL) while remaining unchanged when chemotherapy was supplemented with probiotics (15.7%). *Bacteroidetes* were also under-represented in PRO vs CTRL (4.6% vs. 9.1%). On the other hand, *Actinobacteria*, which were very poorly represented in CTRL (0.056%) and GEM + nab-PTX (0.077%), became strongly enriched in probiotics-supplemented mice (4.1% in PRO and 10.6% in GEM + nab-PTX + PRO). Finally, *Tenericutes*, which were over-represented in chemotherapy-treated mice (30.7%), were even significantly reduced in mice receiving combined treatment (16.4%) with respect to CTRL (23.4%). At the family level (Fig. 5D), when compared to CTRL, GEM + nab-PTX treatment caused an increase in *Anaeroplasmataceae* (13.0% vs. 9.2%) and a decrease in *Lactobacillaceae* (0.004% vs. 0.101%), *Oscillospiraceae* (0.897% vs. 0.945%), *Porphyromonadaceae* (0.071% vs. 0.152%), and *Ruminococcaceae* (7.0% vs. 16.7%). In the comparison between PRO and CTRL, *Clostridiaceae* (11.1% vs. 8.1%), *Oscillospiraceae* (1.4% vs. 0.9%), *Porphyromonadaceae* (0.689% vs. 0.152%), and *Propionibacteriaceae* (0.097% vs. 0.004%) were over-represented whereas *Peptococcaceae* (0.090% vs. 0.291%) and *Ruminococcaceae* (9.5% vs. 16.7%) were less abundant. With respect to CTRL, the combined GEM + nab-PTX + PRO treatment resulted in a lower content of *Acholeplasmataceae* (0.809% vs. 1.2%), *Anaeroplasmataceae* (6.8% vs. 9.2%), *Erysipelotrichaceae* (0.144% vs. 0.188%), *Lactobacillaceae* (0.007% vs. 0.101%), *Oscillospiraceae* (0.852% vs. 0.945%), *Peptococcaceae* (0.125% vs. 0.291%) and *Ruminococcaceae* (7.6% vs. 16.7%) and in a higher content of *Porphyromonadaceae* (1.7% vs. 0.152%), *Prevotellaceae* (0.203% vs. 0.082%) and *Propionibacteriaceae* (0.172% vs. 0.004%). Similarly, combining chemotherapy with probiotics also significantly increased *Porphyromonadaceae* (1.7% vs. 0.071%) and *Propionibacteriaceae* (0.172% vs. 0.000%) with respect to chemotherapy alone. Among the significant changes observed at the genus level (Fig. 5E), all three experimental treatments, compared to untreated CTRL, caused an increase in *Anaerocolumna* (0.262% in GEM + nab-PTX, 0.301% in PRO, 0.283% in GEM + nab-PTX + PRO vs. 0.090% in CTRL) and *Hespellia* (0.179% in GEM + nab-PTX, 0.289% in PRO, 0.240 in GEM + nab-PTX + PRO vs. 0.021% in CTRL) and a decrease in *Caproiciproducens* (0.690% in GEM + nab-PTX, 1.1% in PRO, 0.709% in GEM + nab-PTX + PRO vs. 2.1% in CTRL), *Desulfatomaculum* (0.124% in GEM + nab-PTX, 0.049% in PRO, 0.061% in GEM + nab-PTX + PRO vs 0.247% in CTRL), and *Marvinbryantia* (0.263% in GEM + nab-PTX, 0.205% in PRO, 0.262% in GEM + nab-PTX + PRO vs 1.4% in CTRL). Both GEM + nab-PTX and GEM + nab-PTX + PRO, when compared to CTRL, recorded a lower abundance of *Clostridium* (5.8% and 4.6% respectively vs. 6.8%) and *Lactobacillus* (0.004% and 0.005% respectively vs. 0.08%); chemotherapy alone also decreased *Parabacteroides* content (0.037% vs 0.096% in CTRL), but this decrease was reversed by the combined treatment (0.288%). Supplementing chemotherapy with probiotics, moreover, with respect to chemotherapy alone also produced a significant decrease in *Acholeplasma* (0.585% vs. 1.2%) and *Anaeroplasma* (1.2% vs. 2.2%) together with an increase in *Oscillospira* (1.3% vs. 0.485%) and *Porphyromonas* (0.155% vs. 0.007%). Finally, of note, mice that received PRO treatment alone in comparison to CTRL animals showed a significant enrichment in *Blautia* (0.216% vs. 0.171%) and *Hungatella* (0.105% vs. 0.041%).

**Fig. 6:**
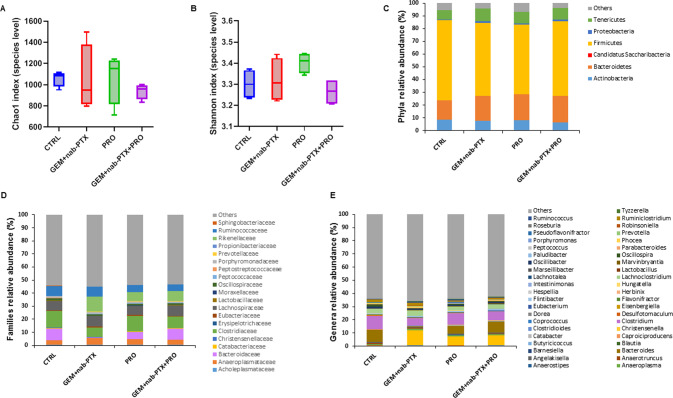
Effect of chemotherapy and/or probiotic treatment on gut microbiota alpha-diversity and taxonomic composition in PANC-1 bearing mice. Boxplots representing species-level richness expressed by Chao1 index (**A**) and diversity expressed by Shannon Index (**B**) in the four experimental groups. Data are expressed as means of four fecal pools per group. Stacked barplots showing the mean relative abundance of gut bacteria at the phylum (**C**), family (**D**), and genus (**E**) level. Data are expressed as means of four fecal pools per experimental group.

Regarding PANC-1 bearing animals, a total of 1,399,611 quality-filtered read pairs were obtained, yielding an average of 87,475.7 read pairs per sample. No significant difference among the experimental groups emerged for either Chao1 (Fig. 6A) and Shannon index (Fig. 6B).

The only significant changes observed among the phyla (Fig. 6C) were the decreased abundance of *Firmicutes* in GEM + nab-PTX compared to CTRL (57.2% vs. 62.8%), and the increase in *Proteobacteria* in both GEM + nab-PTX (1.0%) and PRO (0. 858%) groups compared to CTRL (0.457%). At the family level (Fig. 6D), when compared to CTRL, in all three treatment groups *Clostridiaceae* were decreased (7.2% in GEM + nab-PTX, 11.5% in PRO, 8.6% in GEM + nab-PTX + PRO vs. 12.9% in CTRL) while *Rikenellaceae* were increased (11.3% in GEM + nab-PTX, 6.6% in PRO, 7.5% in GEM + nab-PTX + PRO vs. 0.038% in CTRL). Chemotherapy treatment, with respect to CTRL, also caused increases in *Acholeplasmataceae* (5.3% vs. 3.3%) and *Erysipelotrichaceae* (0.114% vs. 0.046%), which were reversed by probiotics co-treatment (3.8% and 0.046, respectively), and reductions in *Oscillospiraceae* (1.2% vs. 1.9%), *Bacteroidaceae* (0.092% vs. 9.3%) and *Lactobacillaceae* (0.038% vs. 0.281%), which were abolished by probiotics supplementation, too (1.3%, 8.6% and 0.298%, respectively). Finally, *Ruminococcaceae* were under-represented in both PRO (5.5%) and GEM + nab-PTX + PRO (5.2%), when compared to CTRL (7.7%). Among the significantly different genera (Fig. 6E), in all three treated groups with respect to untreated CTRL, *Alistipes* (11.2% in GEM + nab-PTX, 6.5% in PRO, 7.5% in GEM + nab-PTX + PRO vs 0.024% in CTRL), *Dorea* (0.066% in GEM + nab-PTX, 0.080% in PRO, 0.069% in GEM + nab-PTX + PRO vs. 0.040% in CTRL) and *Peptococcus* (0.081% in GEM + nab-PTX, 0.073% in PRO, 0.045% in GEM + nab-PTX + PRO vs. 0.015% in CTRL) were enriched whereas *Clostridium* (5.6% in GEM + nab-PTX, 8.8% in PRO, 6.9% in GEM + nab-PTX + PRO vs 10.1% in CTRL), *Oscillospira* (0.627% in GEM + nab-PTX, 0.854% in PRO, 1.0% in GEM + nab-PTX + PRO vs. 2% in CTRL), *Robinsoniella* (0.078% in GEM + nab-PTX, 0.082% in PRO, 0.105% in GEM + nab-PTX + PRO vs. 0.194% in CTRL), and *Marseillibacter* (0.022% in GEM + nab-PTX, 0.027% in PRO, 0.033% in GEM + nab-PTX + PRO vs. 0.073% in CTRL) were reduced. In addition, GEM + nab-PTX chemotherapy, in comparison to CTRL, caused a drop in *Anaerocolumna* (0.122% vs. 0.210%), *Bacteroides* (0.07% vs. 9.2%), *Desulfatomaculum* (0.055% vs. 0.153%), *Hespellia* (0.020% vs. 0.096%), *Hungatella* (0.017% vs. 0.152%), *Lactobacillus* (0.036% vs. 0.268%), and an increased in *Caproiciproducens* (0.415% vs. 0.120%), which were all reversed by probiotics co-administration (0.142%, 8.5%, 0.113%, 0.077%, 0.055%, 0.293%, and 0.138%, respectively). Finally, mice that received PRO treatment alone in comparison to CTRL animals showed a significant enrichment in *Coprococcus* (0.061% vs. 0.016%) and *Prevotella* (0.247% vs. 0.144%), together with a decrease in *Anaerotruncus* (0.340% vs. 0.554%), *Eisenbergiella* (0.263% vs. 0.311) and *Phocea* (0.329% vs. 0.469%).

All the changes described so far in either BxPC-3 and PANC-1 bearing mice were statistically significant (*p* < 0.05).

### Effect of chemotherapy and/or probiotic administration on animal serum metabolomics

**Fig. 7:**
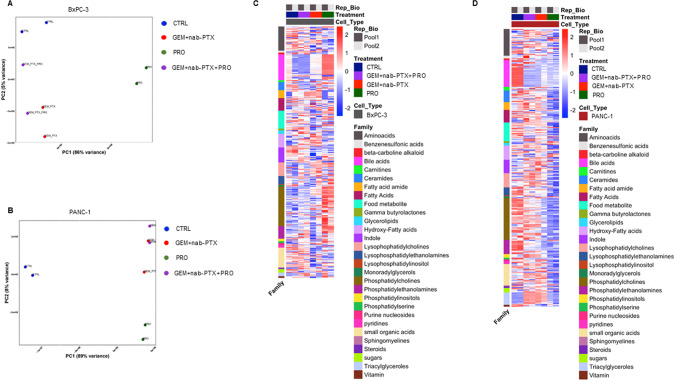
Effect of chemotherapy and/or probiotic treatment on serum metabolomics in PDAC mice. Score plot from PCA model calculated on the relative concentrations (as measured by mass spectrometry peak intensity) of the serum significantly different compounds (ANOVA, *p* < 0.05) in the four treatment groups in BxPC-3 bearing mice (**A**) and in PANC**-**1 bearing mice (**B**). Heatmap representation of the serum significantly different metabolites grouped by classes of compounds (ANOVA, adj *p* < 0.05) in the four groups in BxPC-3 bearing mice (**C**) and in PANC-1 bearing mice (**D**). Two serum pools per experimental group were assayed.

Figure 7A represents the score plot from a PCA model calculated on 9406 features derived from the four chromatographic columns in both positive and negative ionization mode, from two serum pools per experimental condition in BxPC-3 xenografted mice. In the heatmap in Fig. 7C the 281 out of 9406 metabolites significantly different among the four treatment groups are represented, grouped by families of compounds. It can be easily seen that probiotic treatment alone (PRO) remarkably impacts on lipid metabolism, inducing a strong increase in bile acids and in a number of phosphatidylcholines and phosphatidylethanolamines, as compared to other experimental conditions. Moreover, a number of amino acids including glutamic acid, aspartic acid, threonine and serine were significantly decreased in PRO vs. CTRL, together with the serine derivative choline.

Concerning PANC-1 bearing mice, Fig. 7B shows the score plot from a PCA model calculated on 12 931 features derived from the four chromatographic columns in both positive and negative ionization mode, from two serum pools per experimental group. Three hundred and twelve out of all these compounds were found significantly changed among the treatment groups (Fig. 7D). Unlike BxPC-3 bearing mice, in animals transplanted with PANC-1 all three treated groups (GEM + nab-PTX, PRO and GEM + nab-PTX + PRO) showed a sharp decline in serum levels of bile acids with respect to CTRL. In addition, treatment with probiotics alone promoted a decrease in several compounds belonging to the families of phosphatidylcholines and phosphatidylethanolamines, together with some phosphatidylserines, purines and pyridines. On the other hand, the serum of mice receiving chemotherapy, both GEM + nab-PTX and GEM + nab-PTX + PRO groups, was enriched with a number of triacylglycerols.

## Discussion

We previously observed that a specific probiotic formulation was capable of producing positive effects on tumor histology and on some signs of gemcitabine-related toxicity [10] in a xenografted mouse model of PDAC. However, since pancreatic cancers are highly heterogeneous [13, 14] and since gemcitabine monotherapy is no longer the treatment of choice for advanced unresectable disease [6], we investigated the effects of supplementing probiotics to mice injected with BxPC-3 or PANC-1 cells subjected or not to gemcitabine+nab-paclitaxel therapy. A hallmark of PDAC is the deposition of abundant stroma surrounding the tumor, contributing to its malignant phenotype and hindering drug delivery [15, 16]. Probiotics administration in our experimental models resulted in looser collagen fibers compared with control, with more pronounced effects in BxPC-3 carriers. This observation was in line with previous studies which demonstrated a diminished collagen deposition following probiotic supplementation, although in different pathological conditions [17, 18]. Interestingly, probiotics administration in single treatment or in combination with chemotherapy notably induced double-strand DNA breaks and apoptosis in tumor tissues. This observation was in agreement with a number of in vitro studies, included our previous paper [10], which demonstrated the ability of probiotics (or their cell-free supernatants) to induce apoptosis in cancer cells through different mechanisms. Probiotics, indeed, have been shown to promote the expression of pro-apoptotic genes and to lower that of anti-apoptotic ones, to increase caspase activity, to alter chromatin stability through the production of reactive oxygen species and lipid peroxidation, to induce DNA fragmentation, to promote cytochrome c release from mitochondria [19, 20]. Also cell cycle arrest, which we previously demonstrated to be induced by some probiotics strains in pancreatic cancer cell lines [10], can represent a trigger of apoptosis. Furthermore, similar to what observed in our current study, the outer membrane vesicles from the probiotic *Escherichia coli* Nissle 1917 were shown to promote double-strand breaks in DNA (as confirmed by increased Phospho-H2A.X) of colon cancer cells [21].

In addition, in the case of probiotics and chemotherapy co-administration, the ability of probiotics to loosen the stromal deposition around the tumor could further contribute to increase drug delivery and thus to promote cytotoxicity in cancer cells.

Gemcitabine+nab-paclitaxel treatment is known to produce leukopenia and neutropenia as common adverse effects [6, 22]. Indeed, in BxPC-3 bearing animals, white blood cells and segmented neutrophils were significantly reduced after chemotherapy. Probiotics supplementation, however, prevented leukopenia. Moreover, probiotics were also able to recover the significant drop in platelet count caused by chemotherapy and to increase the plateletcrit, in good agreement with previous findings in animals treated with gemcitabine alone [10]. These effects in circulating blood were the result of an enhanced hematopoiesis in the bone marrow, consistent with previous studies in which probiotics were found to counteract myelosuppression and immunosuppression [23, 24]. Again in agreement with our previous findings [10], the probiotics mixture produced a beneficial effect on intestinal regenerative potential and epithelial barrier function.

Some differences between the two animal models emerged with respect to the effects of chemotherapy and probiotics on gut microbiota, being PANC-1 tumors less sensitive to both chemotherapy and probiotics. In BxPC-3 carriers, gemcitabine+nab-paclitaxel chemotherapy, as previously observed with gemcitabine monotherapy [10], caused a drop in microbial species richness, which was restored by probiotics supplementation. Similarly, species diversity was significantly increased in both groups of mice administered with probiotics as compared to mice not receiving the blend. On the contrary, the four experimental groups of PANC-1 injected mice showed no difference in richness and diversity among each other. As for taxonomic analysis, few microbial changes were in common between the two animal models, such as the decrease in *Firmicutes* and *Lactobacillaceae* abundance due to chemotherapy. Probiotics administration had different impacts on BxPC-3 and PANC-1 bearing mice, causing in the former a strong enrichment in beneficial *Actinobacteria* and *Propionibacteriaceae*, while restoring in the latter basal levels of *Bacteroidaceae* and *Lactobacillaceae* heavily decreased by chemotherapy.

Remarkable differences among the two murine models were revealed by the metabolomics analysis of serum samples too. In particular, bile acids, phosphatidylcholines and phosphatidylethanolamines which considerably increased upon probiotics in BxPC-3 bearing mice were instead reduced in all three treatment groups (probiotics included) in the serum of PANC-1 mice. In BxPC-3 carriers, moreover, probiotics treatment induced a drop in some amino acids and in choline, consistently with previous findings. As already discussed, a lower availability of these molecules as important energy sources for biosynthetic processes and proliferation may represent a metabolic disadvantage for cancer cells [10, 25–27].

All the differences observed in this study between the two animal models are not unexpected since it is well known that pancreatic cancer is highly heterogeneous [13, 14] and that BxPC-3 and PANC-1 cell lines are quite different from each other for both genotypic and phenotypic aspects [11]. In more detail, BxPC-3 cells display an epithelial phenotype whereas PANC-1 show a more mesenchymal-like one, which likely contribute to their different sensitivity to gemcitabine [14, 28]. This could explain why gemcitabine+nab-paclitaxel chemotherapy differently impacted on the two mouse models in the present study. In addition, many differences were also demonstrated at metabolic level between the two cell lines, with particular focus on carbohydrate and lipid metabolism [29].

Finally, since we observed that gemcitabine alone [10, 30] or combined with nab-paclitaxel, affects gut microbiota in mouse models of PDAC and since it was reported that chemotherapy reshapes the gut microbiota in human patients suffering from different types of cancers [31–34], often promoting an overgrowth of inflammatory *Proteobacteria* [31, 33], we plan to investigate, in the next future, the microbiota composition of PDAC patients before and after receiving chemotherapy.

Taken together all the observations in the current study strongly encourage to investigate on possible benefits of supplementing selected probiotics to PDAC patients under chemotherapy.

## Materials and methods

### Animals, experimental design, and samples collection

The experimental protocol on animals was approved by the Italian Ministry of Health (approval number 959/2020-PR). Female nude BALB/c mice (5–8 weeks old) were purchased to establish the subcutaneous xenograft models of pancreatic cancer, by injecting 4.5 × 10^6^ BxPC-3 or PANC-1 human cells in the animal flank. When tumors reached an average volume of 100 mm^3^, mice bearing each of the two cell lines were randomized into the following experimental groups (*n* = from 6 to 9/group): CTRL (control, administered with saline solution), GEM + nab-PTX (50 mg/kg gemcitabine and 5 mg/kg nab-Paclitaxel twice a week each, intraperitoneally), PRO (probiotics mixture by oral gavage five consecutive days/week, as previously described [10], GEM + nab-PTX + PRO (50 mg/kg gemcitabine and 5 mg/kg nab-Paclitaxel twice a week each i.p., plus probiotics mixture by oral gavage five consecutive days/week). The probiotics mixture administered to mice was previously described in detail elsewhere [10], but essentially contained *Bifidobacterium breve* SGB01, *Bifidobacterium bifidum* SGB02, *Lactobacillus kefiri* SGL13, *Lactobacillus plantarum* SGL07, *Lactobacillus salivarius* SGL03, and *Lactobacillus reuteri* SGL01 completed with inulin and lactoferrin. All the treatments were performed for two weeks, during which animals had free access to food and water and were monitored daily for signs of suffering. Body weight and tumor volume were measured twice/week. The experimental design was schematically represented in Fig. 1A.

At the end of the protocol, a whole blood specimen was collected from the mandibular plexus, which was partly used for blood cell count and partly centrifuged to obtain serum for both biochemical and metabolomics analyses. Fresh fecal pellets were also harvested from clean cages and stored at −80 °C until use. Mice were euthanized by CO_2_ inhalation, then tumors and intestines were explanted, formalin-fixed and paraffin-embedded for histological analyses. In addition, animal femoral bones were also explanted, formalin-fixed, decalcified and paraffin-embedded for histological examination of bone marrow.

### Histological and immunohistochemical analyses

Mouse tissues were fixed in 4% paraformaldehyde and were paraffin-embedded with Diapath automatic processator. Subsequently formalin-embedded tissues were cut into 4 µm sections and routinely stained with Haematoxylin and Eosin (H&E) (Diapath, Italy) according to standard protocol and samples were mounted in Eukitt (Bio-Optica, Italy) for histopathological analysis, performed by pathologists with specific expertize in murine pathology.

In order to evaluate the stromal remodeling, Picrosirius red staining (Scy Tek Lab, SRS-IFU) was carried out and allowed to appraise arrangement and packing of collagen I and III fibers collagen.

Histological visualization of intestinal mucins was performed using Alcian Blue pH 2.5/PAS staining (Bioptica 04-163802).

For immunohistochemical analysis, paraffin was removed with xylene and the sections were rehydrated in graded alcohol. Antigen retrieval was carried out using preheated target retrieval solution for 30 min. Tissue sections were blocked with FBS serum in PBS for 60 min and incubated overnight with the following primary antibodies: Ki67 (Thermoscientific, Italy, MA5-14520), Phospho-γH2A.X (Abcam, Italy, ab1174), Arginase-1 (GeneTex, USA, California, GTX109242), Apoptosis-Inducing Factor or AIF (Cell Signaling, USA, Massachusetts, 5318), Myeloperoxidase (Abcam, ab208670), CD41 (Abcam, ab181582), PAX5 (Abcam, ab109443), TER119 (Abcam, ab91113), and Endomucin (Abcam, ab106100). The antibody binding was detected using a polymer detection kit (GAM/GAR-HRP, Microtech, Italy) followed by a diaminobenzidine chromogen reaction (Peroxidase substrate kit, DAB, SK-4100; Vector Lab, USA, California). All sections were counterstained with Mayer’s hematoxylin and visualized using a bright-field microscope. For immunofluorescence, tumor sections were incubated over-night with an antibody against Alpha-SMA (Sigma-Aldrich, Italy, A5228) and then incubated with anti-mouse Alexa Fluor 488 (1:200, Molecular Probes, Invitrogen Life Technologies, Grand Island, New York) for 1 h at room temperature, mounted with a PBS/glycerol solution and examined under a Leica TCS SP2 confocal microscope.

A custom Fiji [35] plugin was used to extract the area positive for the Picrosirius positive (red/purple) or the αSMA positive area. In order to segment Picrosirius positive area, the channels of the acquired RGB (red, green and blue) were separated. Given the red/purple nature of the Picrosirius labeling the green component of the RGB image shows a negative image of the Picrosirius positive staining and was used therefore used for segmentation. The green channel of each image was first inverted and subsequently segmented with the Moments ImageJ [36] thresholding method. The resulting segmented image was subjected to an erosion step, follow by a ‘remove outliers’ step (radius 10, bright) and by a final dilation step. After the segmentation, the positive area was measured. To segment the αSMA positive area, a median filter (radius 2) was first applied to the images. Then, the Li [37] thresholding method of Fiji was applied. The segmented image was finally measured to assess the extent of αSMA positive area.

### Fecal DNA isolation, 16S rRNA gene sequencing and taxonomic analysis

Frozen fecal pellets underwent microbial DNA isolation following the protocol provided by the QIAamp FAST DNA Stool Mini Kit (Qiagen, Italy). Universal primers from Klindworth et al. [38] were used to amplify the V3-V4 hypervariable region of the bacterial 16 S rRNA gene. Libraries were prepared following the Illumina 16 S Metagenomic Sequencing Library Preparation protocol, as previously described [39], pooled at equimolar ratio and paired-end sequenced (2 × 300 bp cycles) on an Illumina MiSeq platform (Illumina Inc). Sequence data generated as FASTQ files are deposited in the Arrayexpress repository under accession code E-MTAB-12171.

De-multiplexed FASTQ files were imported into the 16S Metagenomics GAIA 2.0 platform (Sequentia Biotech, Barcelona, Spain, 2017), in which quality check and trimming were performed through FastQC and BBDuk. High quality reads were then mapped with BWA-MEM against a custom NCBI-based database (released on 2020), to obtain taxonomic classification. For the compositional analysis, an abundance filter was applied to retain only OTUs > 0.1% of the total abundance per sample, in at least one of the analyzed samples. OTUs with lower abundance or unclassified were indicated as “Others”.

### Serum metabolomics

Pooled sera were obtained from each of the four experimental groups of mice (CTRL, GEM + nab-PTX, PRO and GEM + nab-PTX + PRO) xenografted with BxPC-3 or PANC-1 cells. Two pools per condition were subjected to four types of metabolome profiling: (i) small and highly polar metabolites with use of HILIC chromatographic column, (ii) medium polar metabolites with use of RP C18 chromatographic column; (iii) lipidome with use of RP C8 chromatographic column, and (iv) zwitterionic compounds with use of pZIC-HILIC chromatographic column. The Waters Ostro 96 well plates were used to extract metabolites from serum and quality control (QCs) samples. Two fractions were collected during extraction procedure: small molecule fraction eluted with cold acetonitrile with 1% of formic acid, and lipid and phospholipid fraction retained on well plate filter, and eluted with mix 4.5:4.5:1 chloroform: methanol: trimethylamine. Details regarding extraction procedure together with description of mobile phases and mass spectrometry conditions are presented in supplementary Figs. S2 and S3. Annotation of statistically significant metabolites was performed against in-house library, while remaining unknown compounds underwent the meticulous manual structure elucidation process. Serum samples were fully randomized for extractions and injections steps. Double injection of QC sample was set every eight study samples along each queue.

### Processing of metabolomics data

The raw.wiff files were converted into.abf with use of ABF Converter and analyzed by MS-DIAL software. The MS-DIAL analyses were performed separately for each of the four metabolomics assays, separately for positive and negative ionization modes. For each processing run, blanks, QC and all study samples were used. For normalization of samples, the LOWESS approach was used, available within MS-DIAL software. The statistical significance (ANOVA) was calculated separately for each of cell lines PANC-1 and BxPC-3 in R environment. As consequence two heatmaps were created considering annotated metabolites with adj *p* val < 0.05.

### Statistics

As for tumor volumes, alpha diversity indices and blood analyses, Student t-test was used to compare means in pairwise comparisons. Differential abundance analysis of taxonomic data was performed using DESeq2 statistics. Results were considered significant when *p* < 0.05.

ANOVA statistical test corrected for FDR was applied to analyze metabolomics data. Results were considered significant when adj *p* < 0.05.

## Supplementary information

Supplementary Figure Legends

Figure S1.

Figure S2

Figure S3

Authors agreement to additional authorship

## Data Availability

Sequence data generated as FASTQ files are deposited in the Arrayexpress repository under accession code E-MTAB-12171. Data of the present study will be available from the corresponding author upon reasonable request.

## Materials availability

Sequence data generated as FASTQ files are deposited in the Arrayexpress repository under accession code E-MTAB-12171. Materials of the present study will be available from the corresponding author upon reasonable request.
