# Supplementary material for: Counteracting gemcitabine+nab-paclitaxel induced dysbiosis in KRAS wild type and KRASG12D mutated pancreatic cancer in vivo model
Source: Cell Death Discov. 2023 Apr 5;9:116. doi: 10.1038/s41420-023-01397-y (PMC10076501; doi:10.1038/s41420-023-01397-y)
Supplement: Supplementary file 1 — Supplementary Figure Legends [file 41420_2023_1397_MOESM1_ESM.docx]

**Supplementary figures’ legends**

**Figure S1. Histochemical and immunohistochemical quantifications on mice tumor sections.**

Quantification of Picrosirius Red staining in BxPC-3 **(A)** and PANC-1 **(D)** mice tumor tissues. At least 9 fields per experimental group were analysed.

Quantification of α-SMA staining in BxPC-3 **(B)** and PANC-1 **(E)** mice tumor tissues. At least 9 fields per experimental group were analysed.

Quantification of Phospho-H2A.X staining in BxPC-3 **(C)** and PANC-1 **(F)** mice tumor tissues. At least 9 fields (at 40X magnification) per experimental group were analysed.

Pairwise comparisons were performed by t-test and differences were considered significant when *p* < 0.05 (*), *p* < 0.01 (**) or *p* < 0.001 (***).

**Figure S2. Schematic representation of sample preparation for metabolomics analysis.**

**Figure S3. Schematic representation of chromatographic and mass spectrometry conditions used for metabolomics analysis.**
