# Supplementary figures and images for: Counteracting gemcitabine+nab-paclitaxel induced dysbiosis in KRAS wild type and KRASG12D mutated pancreatic cancer in vivo model
Source: Cell Death Discov. 2023 Apr 5;9:116. doi: 10.1038/s41420-023-01397-y (PMC10076501; doi:10.1038/s41420-023-01397-y)

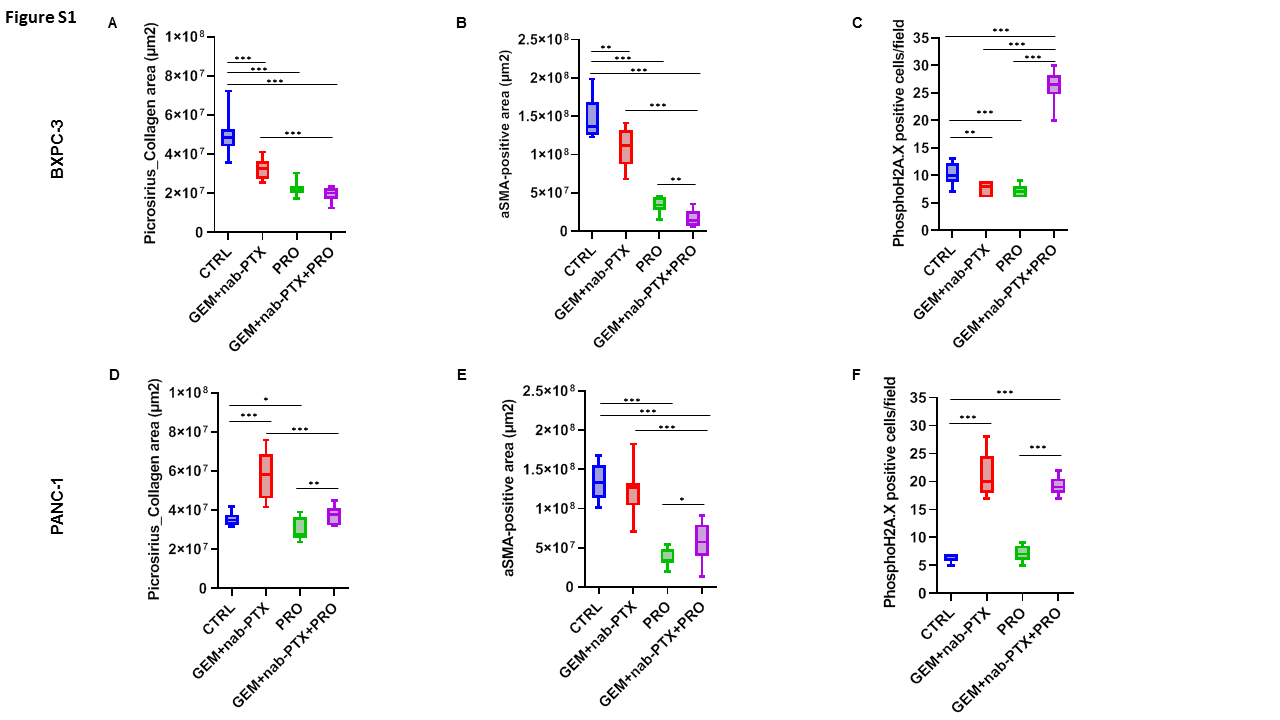

Supplement: Supplementary file 2 — Figure S1. [file 41420_2023_1397_MOESM2_ESM.tif]

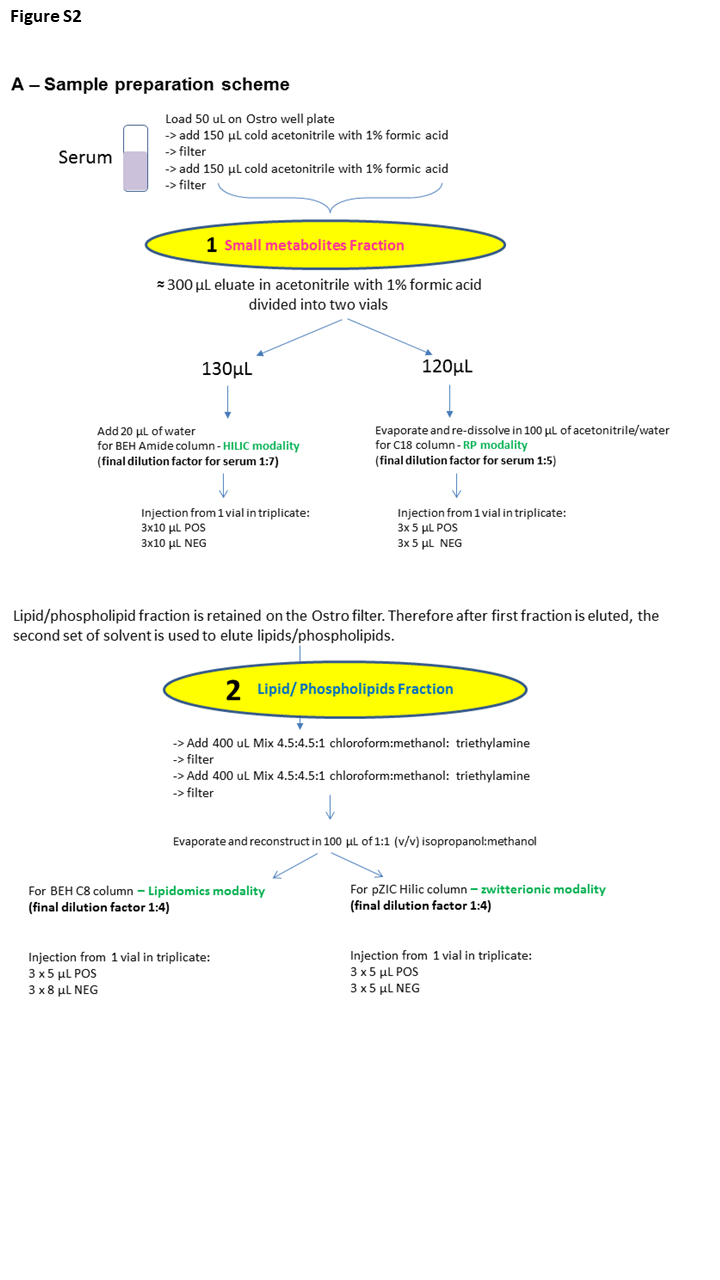

Supplement: Supplementary file 3 — Figure S2 [file 41420_2023_1397_MOESM3_ESM.tif]

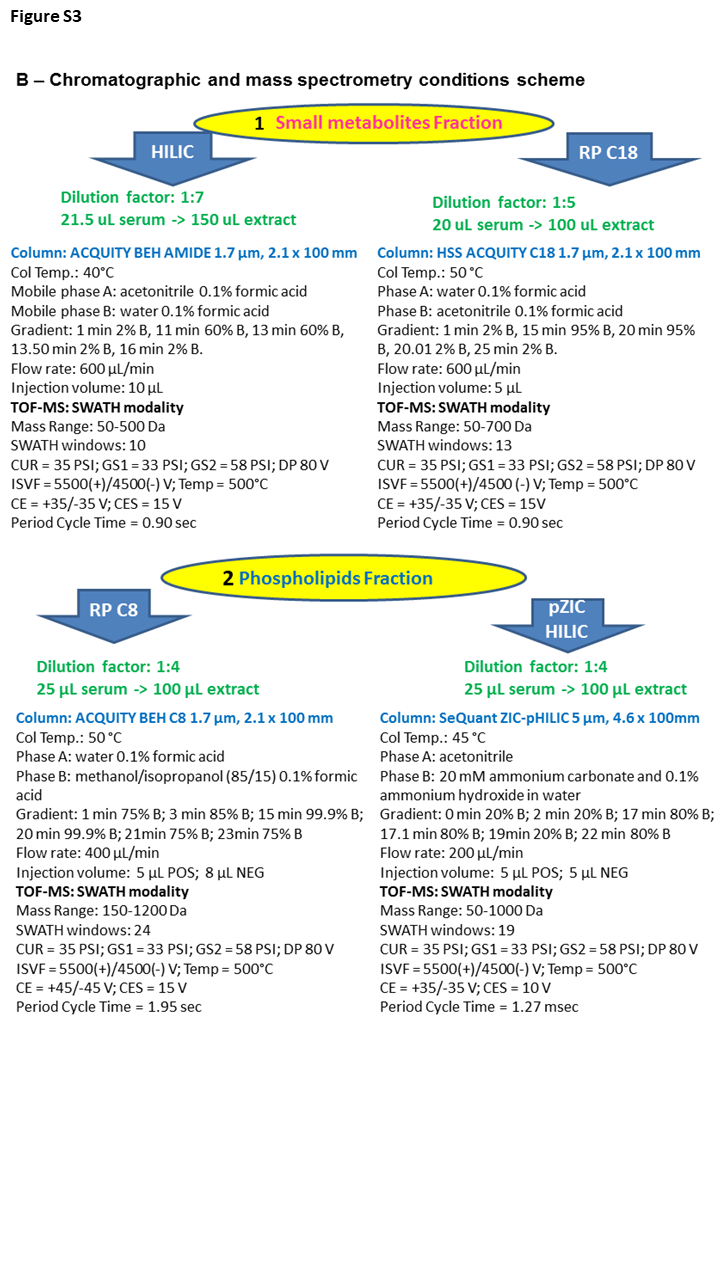

Supplement: Supplementary file 4 — Figure S3 [file 41420_2023_1397_MOESM4_ESM.tif]
