# Supplementary material for: Counteracting gemcitabine+nab-paclitaxel induced dysbiosis in KRAS wild type and KRASG12D mutated pancreatic cancer in vivo model
Source: Cell Death Discov. 2023 Apr 5;9:116. doi: 10.1038/s41420-023-01397-y (PMC10076501; doi:10.1038/s41420-023-01397-y)
Supplement: Supplementary file 5 — Authors agreement to additional authorship [file 41420_2023_1397_MOESM5_ESM.pdf]

Federica Pisati
a Valerio, me, Annacandida, Andolfo, Marynka, claudio.tripodo, Carmelapia, Francesco, Renato, bellini.edoardo, Tiziana, beatricebelmonte@virgilio.it

1 mar 2023, 16:43 (16 ore fa)

Dear valerio
I agree to add Dr. Orsenigo Fabrizio as a co-author in this work CDDISCOVERY-22-5648R
Federica Pisati

--
FP

Andolfo Annapaola
a bellini.edoardo, Valerio, me, Annacandida, Marynka, claudio.tripodo, Carmelapia, Francesco, Renato, Tiziana, beatricebelmonte@virgilio.it, Federica

mer 1 mar, 18:13 (15 ore fa)

Dear Valerio,
I agree to add Dr. Orsenigo Fabrizio as a co-author in this work CDDISCOVERY-22-5648R
Regards,
Annapaola Andolfo

Annapaola Andolfo, Ph.D.
ProMeFa, Proteomics and Metabolomics Facility
Dibit 2, San Gabriele 2 building, C1 area, 4th floor
San Raffaele Scientific Institute
Via Olgettina, 60
20132 Milan, Italy
Ph: +39-02-26432714/2253/4928
e-mail: andolfo.annapaola@hsr.it
web site: https://research.hsr.it/en/core-facilities/promefa.html

---

PERRI H
a Valerio, me, Annacandida, Andolfo, Marynka, Federica, claudio.tripodo, Carmelapia, Renato, bellini.edoardo, Tiziana, beatricebelmonte

07:18 (2 ore fa)

Dear All,

I agree to add Dr. Orsenigo Fabrizio as a co-author in this work CDDISCOVERY-22-5648R.

Best regards,

Dr. Francesco Perri

Marynka Ulaszewska
a bellini.edoardo, Valerio, me, Annacandida, Andolfo, Federica, claudio.tripodo, Carmelapia, Francesco, Renato, Tiziana, beatricebelmonte@virgilio.it

08:41 (40 minuti fa)

Dear Valerio,
I agree to add Dr. Orsenigo Fabrizio as a co-author in this work CDDISCOVERY-22-5648R

Thank you for your attention
kind regards
Marynka Ulaszewska

beatricebelmonte@virgilio.it 08:52 (29 minuti fa)
a Valerio, me, Annacandida, Andolfo, Marynka, Federica, claudio.tripodo, Carmelapia, Francesco, Renato, bellini.edoardo, Tiziana

Dear Valerio,

I and Claudio are agree to add Dr. Orsenigo Fabrizio as a co-author in this work CDDISCOVERY-22-5648R.

Regards,

Beatrice and Claudio

Concita Panebianco <panebianco.c@gmail.com> 09:09 (35 minuti fa)
a pazienza_valerio

Dear Valerio,

I agree to add Dr. Orsenigo Fabrizio as a co-author in this work CDDISCOVERY-22-5648R.
Regards,

Concetta Panebianco

--
Dr. Concetta Panebianco, Ph.D
Laboratory of Research, Gastroenterology Unit
IRCCS "Casa Sollievo della Sofferenza" Hospital
71013 San Giovanni Rotondo-FG
Phone +39.0882.416281
Fax +39-0882.410271

Carmelapia Ferro <carmelapia.ferro@outlook.it> 09:33 (9 minuti fa)
a Valerio, me

inglese > italiano Traduci messaggio Disattiva per: inglese

Dear Valerio,

I agree to add Dr. Orsenigo Fabrizio as a co-author in this work CDDISCOVERY-22-5648R.

Regards,

Carmela

Da: Annacandida Villani <villaniannacandida@yahoo.it>
A: Valerio Pazienza <pazienza_valerio@yahoo.it>
Inviato: giovedì 2 marzo 2023 alle ore 09:07:16 CET
Oggetto: Re: Richiesta integrazione a: Decision letter for CDDISCOVERY-22-5648R: Accepted in Principle

Dear Valerio,
I agree to add Dr. Orsenigo Fabrizio as a co-author in this work CDDISCOVERY-22-5648R
Annacandida

Dr. Annacandida Villani
Laboratory of Research, Gastroenterology Unit
IRCCS "Casa Sollievo della Sofferenza" Hospital
71013 San Giovanni Rotondo-FG
Phone: +39 0882416281

----- Messaggio inoltrato -----
Da: Fabrizio Orsenigo <fabrizio.orsenigo@ifom.eu>
A: Valerio Pazienza <pazienza_valerio@yahoo.it>; Federica Pisati <federicapisati@gmail.com>
Inviato: giovedì 2 marzo 2023 09:47:06 CET
Oggetto: Re: Richiesta integrazione a: Decision letter for CDDISCOVERY-22-5648R: Accepted in Principle

I agree to be included as co-author of the above mentioned manuscript.

Regards

Fabrizio Orsenigo

b
bellini.edoardo 10:07 (0 minuti fa)
a Marynka, Annacandida, Andolfo, Federica, claudio.tripodo, Carmelapia, Francesco, Renato, Tiziana, beatricebelmonte@virgilio.it, Valerio, me

inglese > italiano Traduci messaggio Disattiva per: inglese ×

I completely agree to add Orsenigo Fabrizio as coauthor of paper with ID-code : CDDISCOVERY-22-5648R

Edoardo Bellini

Computational Biology and Bioinformatics
Center for Omics Sciences @OSR (COSR)
DIBIT2, A3, Piano 3, room 15
Stem Cells and Neurogenesis Unit
Division of Neuroscience
San Raffaele Scientific Institute
Via Olgettina 58, 20132 Milan, Italy

----- Messaggio inoltrato -----
Da: Tiziana Pia Latiano <latianotiziana@gmail.com>
A: Valerio Pazienza <pazienza_valerio@yahoo.it>
Inviato: giovedì 2 marzo 2023 11:03:06 CET
Oggetto: Re: Richiesta integrazione a: Decision letter for CDDISCOVERY-22-5648R: Accepted in Principle

Dear All,

I agree to add Dr. Orsenigo Fabrizio as a co-author in this work CDDISCOVERY-22-5648R.

Best regards,

Dr. Tiziana Pia Latiano

•••

Rispondi Inoltra

Renato Lombardi <r.lombardi@operapadrepio.it> 13:22 (29 minuti fa)
a bellini.edoardo, Marynka, Annacandida, Andolfo, Federica, claudio.tripodo, Carmelapia, f.perri, Tiziana, beatricebelmonte@virgilio.it, Valerio, me

Dear Valerio,
I agree to add Dr. Fabrizio Orsenigo as a co-author in this work CDDISCOVERY-22-5648R
regards
renato lombardi
